# Cottage Asylums:—A Sequel

**Published:** 1861-07

**Authors:** W. A. F. Browne

**Affiliations:** One of the General Board of Commissioners in Lunacy for Scotland.


### Art. XI.—COTTAGE ASYLUMS:—A SEQUEL.

By W. A. F. Browne, One of the General Board of Commissioners in Lunacy for Scotland.

"The Gheel system is not one that I should like to see followed in this country." —*Speech by Dr. Conolly at a Meeting to promote the Erection of an Asylum for the Middle Classes.—Times, April* 22, 1861.

The preceding observations* upon this subject were intended to remove the difficulties which appeared to surround the conception of a Colony of the Insane; to reduce the proposal of engrafting such a benevolent scheme upon our social system to its real proportions; and to introduce such a modification of the principle as appeared attainable and adaptable to existing institutions in a plain and practical form. The sacred but debateable territory of Gheel shall not be trodden, it shall scarcely be touched again. But, in thinking out the thesis, it was early observed that to any effort to withdraw from their homes, natural position, and individual pursuits and objects, a large body of men, and compulsorily to assemble and retain them together for a common purpose, but one in which they felt no interest, towards which they might entertain a strong repugnance, or which they could not understand, the designation of a colony, at least in its ordinary acceptation, was singularly inappropriate. It was besides palpable that in any humble imitation of an institution which had been the growth of ages, which had grown with the faith of which it may fairly be claimed as a concomitant, if not a result,—like the ivy on mediæval towers—there would be lacking both the religious element, with which it was assimilated, if not identified, and the slow and gradual development by which it was characterized. In endeavouring to secure what was valuable in the experiment, it became obvious that it would be impossible to create at once in working order and with any chance of success, of even similar success, a machinery which had any parallel in history, any analogue in our present polity, any foreshadowing in the aspiration of our boldest philanthropists. It became imperative to seek a precedent; to discover a body of men, who, moved by other impulses than their own wishes or supposed interests, by causes differing altogether from the love of discovery, or adventure, or conquest, the craving for gold or gain, the surplusage of population and the off-casting of the parent hive, such as actuate ordinary emigrants to relinquish their original ties, or some of them; and who had been necessitated

---

* See No. II. of this Journal, p. 213.

to associate together, to submit to new laws and systems, and to act upon new and, in some respects, abstract principles ;—and to obtain from such communities sanction, and encouragement, and suggestions as to the innovation contemplated. Through the analysis many forms of social arrangement passed ; there were German villages in Spain, and French villages in Scotland considered, but in none of these could be recognised the principles or features required ; and yet, in other localities less connected with our race and habits were detectable peculiarities which pointed to a compulsory colony, originated for the benefit and gradual emancipation of its members.

I. It may seem bold and romantic to seek on the steppes of Russia, and among a people whose early architecture was compared by Gibbon* to that of beavers, for support to a modern theory. Yet the Cossacks, who appear mistily on the margin of history about a thousand years ago, when St. Dymphna lived and died, although represented by the Byzantines as a distinct people, repudiated by the Russians, yet claimed as Caucasians, were so increased by the aggregation from other countries and tribes, as to deserve the title of outcasts and fugitives ; lived in a land to which they did not belong ; were subjected to a species of serfdom from which they were liberated in virtue of services rendered, duties performed in relation to a moral standard which they did not erect ; and the professors of a certain faith and allegiance. Yet these hordes—migratory military, robber-like ; these bulwarks of the Russians and Poles against the gulf-stream of Eastern warfare were, in a certain sense, captives, devotees to a cause in which they had no selfish nor personal object ; laboured and fought for freedom and food ; they were celibats ; they were organized and subjected to a system of rule so rigid and omnipotent, as to annihilate individual will and to fuse into one common mass the heterogeneous and stubborn qualities of the most intractable materials. They hated the aborigines among whom they were planted ; they did not amalgamate with them ; they were in some respects superior to them, affording examples of industry and activity in their duties, of cleanliness and comfort in their dwellings. It is true that the descendants of these nomades have become the discoverers and conquerors of vast and valuable tracts in Asia, have undergone, under the shadow of a mighty monarchy, revolutions mimicking in their transition from democracy to tyranny those of distant empires ; that they have uttered their war-cry in the palaces of the capital of the West ; but even now there are preserved on the Don and Dnieper vestiges of the attributes which assimilated them to a settlement where the

---

* Chap. xlii.

inhabitants had no home, no family tie, to which they were confined by circumstances over which they had ceased to exercise control, and in the progress and prosperity of which they were blind, impassive, and not always willing instruments.*

II. It is, however, under a hierocracy that the more complete development of such a colony is discovered. The Church is the centre of the system. It is not the province of such an inquiry to determine either the precise objects, the means resorted to, or the results achieved in the missions of Paraguay; where, in common with the spirit which characterized European society, the sentiment of personal independence and the passion for individual liberty found no entrance. The priests by whom that marvellous organization was perfected were apostles, as far as going forth to propagate the truth with scrip and staff, and remaining penniless; they were martyrs, as watering the soil with their blood; they were philosophers, as leaving to us an example of what faith, order, training may accomplish when exercised by the gifted over the credulous, by the earnest and astute over the simple-minded. All the colonies, or reductions, or towns in which these moral victories were secured over savage wills and passions, were constructed upon the same plan. The dwellings of the inhabitants, or pupils, formed three sides of a square. These were, at first, mere hovels, consisting of a framework of stakes with stones between them, and then plastered over with mud and straw, very closely resembling many of those huts still used by our countrymen in the Highlands. The interior consisted of a single room twenty-four feet square, the door of which admitted light, and served as a chimney. These were erected and repaired by the community. To each couple or family was apportioned one containing a hammock, a few vessels of pottery or gourds, a chest or two, and a few benches. On the fourth side stood the place of worship, close to which were the priests' residences, public workshops, a separate house for widows—all enclosed within quadrangles, the burial ground, and a large garden containing medicinal as well as pot-herbs. The churches were described by those who conceived them to be desecrated by the purpose which they served, as the most spacious and splendid in that part of the world. Their windows of paper or talc shut out the fierce sun, but they glowed with gilding, and pictures, and sculpture, they were fragrant with flowers and sprinkled essences; and the symbols of Christianity spoke to the Indian in a language which even he could understand. The cemetery was, in anticipation of modern taste, planted and bordered with shrubs and flowers; and

---

* Dr. E. D. Clarke, *Travels in Europe, Asia, and Africa; History of Russia*, by Alph. Rabbe, &c.

the dust of the different sexes and ages slept separately in their appointed graves. There was, in a sense, a community of goods. The head of every family was allotted a portion of land whereon to raise maize, potatoes, cotton for himself, by the aid of oxen lent from the common stock. Of this he continued tenant so long as he was able to cultivate it. But at stated times each was called upon to labour in the Tupamba or God's possessions, the produce of which passed into the storehouse for the behoof of the infirm, the sick, of widows and orphans, and for public purposes. The corregidors, alcaldes, &c., by whom the municipal government of these townships was supposed to be carried on resembled that of Spanish towns, but in the missionaries resided the authority and administration; the machinery was ecclesiastical; every act was either dictated by or received its sanction from religion. Two priests, at least, regulated each community; one never left the scene of his labours, but passed from the altar to the workshop; the other, knowing intimately the language and habits of his children, a saint-errant, visited the out-field, gathered in new fruits, ministered to the sick or the sorrowful. Subordinate to these were trained Indian acolytes who watched over and reported the condition, mental, moral, and physical, of every inmate. Holy guilds or confraternities there were for mutual support and communion; that of St. Michael the Archangel for the men, the active, the laborious; that of the Mother of God for females, the pious, and the contemplative. Amusements there were, varied in character, but interpenetrated by the prevailing principles. The music was sacred; the dramas were of the Virgin, the Three Kings of Cologne; dancing was confined to the boys. The separation of the sexes was commenced in the cradle, was scrupulously enforced throughout life, except where intercourse was consecrated by marriage, and carried beyond death. Children were regarded as the property of the commuity, to be moulded, and made, and destined according to the will and model of their religious superiors. While the girls gathered and the women spun cotton, the boys made and mended roads, the men were engaged as masons, carpenters, turners, painters. They cast bells, built organs, and were most dexterous imitators of everything presented to them. Morning was hailed by prayers and mass; the day was divided as in monastic establishments, and each section marched to work in procession, headed by an image of St. Isidore, and chanting hymns and canticles. To labour and to pray were there the only duties and objects of life; and only such children as were designed for public offices were taught to read and write. There were punishments, but, according to the evidence of those who were at once teachers and judges, little crime; years passing without the commission of a deadly sin; restriction and retribution being, in all probability, directed to the confirmation of that inflexible routine, that implicit obedience, which were the characteristics and objects of monachism. Yet there survived generous and noble impulses under what is styled a benumbing influence. These artificial beings opposed the slave trade; they were roused by oppression and interference, defended their reductions, and repulsed the troops of Spain and Portugal. And yet, according to one who lingers lovingly upon some aspects of the system under which they were trained, an Indian was little more advanced at seventy than at seventeen. It may be that this vast organization became practicable on account of the simplicity, the intellectual weakness of its members; it may be that its power was exerted in producing or in perpetuating, while it used and directed, the feebleness and flexibility which it found. It is necessary to repeat that this episode in Christian history is no romance. Neither poet nor painter has added a colour to the picture. It is composed of traits and facts, in which perhaps the Jesuits saw nothing either beautiful or wondrous, nothing more than the cold, sharp outline of their duty, which existed a few generations back, which concerned hundreds of thousands of individuals, and which have left a few faint traces in their descendants. It matters little whether the experiment comprehended merely cannibal caciques and their savage tribes, or dispositions softened and prepared by an extinct civilization, the results would be equally demonstrative of the triumph of system over masses of untrained if not of untractable men, of the formation of a colony upon a basis in which material prosperity was a subordinate, moral benefit and intellectual development were the prominent considerations.*

III. Even when a noble cause is at stake, war and civil commotion loosen and break down the bonds by which society is coerced into industrious habits and regular proceedings, and call forth a class disposed to prolong the licence and the recklessness of the military life; to live upon the liberality or timidity of others rather than by their own exertions. It remains to be seen whether the spirit of modern civilization will, in time of social convulsion, prevent such a result, or force back at once such encroachments within their natural and prescribed limits. There was much simplicity and sameness in the remedies proposed in former times. On several occasions it was advocated that the multitudes of mendicants, and desperadoes, and idlers who remained, like the traces of a tempest, after peace and order had been so far restored, and armies disbanded, should be deported as slaves. This was actually carried into effect, and by an Englishman to boot, in the case of the thousands of youths and maidens

---

*. Southey's *History of Brazil, passim.*

assembled together on the shores of the Mediterranean by the epidemic madness called the children's crusade.*

We find the patriotic political economist, Fletcher of Saltoun, who recoiled from the defence of the country being intrusted to mercenary troops, describing vast numbers of vagrant and lawless and Godless poor, estimated at 200,000, who oppressed and terrified the peaceable and industrious inhabitants. These, or a larger portion of the able-bodied among them, he gravely recommends should be reduced to serfdom under such persons as would undertake to keep and employ them ; or that, for example sake, they should be presented to the State of Venice to serve in the galleys against the common enemy of Christendom. At a conjuncture, after the last European war, when Holland was visited by a similar scourge of marauding mendicancy, philosophers suggested the milder expedient of forming agricultural colonies for the self-maintenance and the training of the poor, and this in a country where beggary and vagrancy are punishable by imprisonment. The principle upon which the hopes of the philanthropists who originated the enterprise, and of the great society which gathered around them, were founded, seems to have been that if the savage can, unaided, compel the earth to yield him sustenance, the indigent civilized man, under the guidance of science and experience, and supplied with capital and instruments, should be able to maintain himself and others. But the objects ultimately in view had a higher range, and comprehended the preservation in the minds of the donors of those feelings which entitle their gifts to be dignified with the name of charity ; and the cultivation in the minds of the recipients of that independence and those habits of regularity and industry which are either incompatible with pauperism, or impart to poverty nobility and virtue. In carrying out this project, several thousands of acres of barren heath were purchased by means of the original subscriptions of the members of the society, upon which able-bodied beggars and paupers were settled with a view to reclaim and render them productive. The land was apportioned into lots of about seven acres, which it was calculated would support a family of six persons ; who were offered a well-built house, education for their children, and medical aid, in return for about 142*l.*, to be advanced by the parish to which the colonists belonged, or from private resources, the payment to be spread over sixteen years, or until the original outlay had been refunded by the exertions of the settler, and until he had become self-supporting. It is germane to the present inquiry to notice that the house and buildings for a family of seven or eight individuals on each colonial farm cost

---

* Michaud, *History of the Crusades.*

about 42*l.*, the furniture and agricultural implements 8*l.*, and that it consisted of one public room fifteen feet by twelve feet, and of four closets, six and a half by six feet, apparently without windows, and sixteen feet in height. These habitations were built of brick made from clay found on the spot, floored with tile or brick, and roofed with reeds, and were placed at regular intervals on the sides of the broad roads by which the settlement is intersected. In 1825 the gross population amounted to 6778, distributed in eight establishments; 416 dwelling houses, six large depots for children and paupers, and 37 large farms and dependencies; 3227 were grouped together in families. The mortality appears to have been nine per cent. This experiment is most fully described in a work published in 1828, by a member of the Highland Society of Scotland, who pronounces it, so far as Fredericksoord is concerned, to have been completely successful.* In 1838 Mr. W. Chambers speaks of these colonies; and as keeping the streets of the Dutch towns free of mendicants and all sorts of disorderly persons.† A most interesting and masterly report, from the pen of Sir John M'Neil, appeared as an Appendix to the "Eighth Report of the Board of Supervision, 1853, on the Free and Pauper Colonies of Holland," and forms the most ample and authentic body of evidence upon the subject. From these observations it appears that not above sixteen or twenty colonists, or one per annum of the whole number, had been able to emancipate themselves during the thirty years past, by paying the debt due to the society, or the annual rent of 4*l.*; that independent tenants beyond the settlement pay the landlord 7*l.* or 8*l.* and live. It is calculated that the debt on each allocation amounts to 2352 florins; but if the improvement of the land be taken into consideration, the value of the house and then the deterioration of that, there would remain an uncovered balance of 800 florins, or 66*l.* 13s. 4d. against the society for each lot, equal to 2*l.* per annum since the commencement; and were the society to wind up its affairs, it would be found insolvent to the amount of 127,952*l.* It is further shown that each pauper costs the Dutch Government 6*l.* 13s. 4d. per annum, and this does not include interest on capital invested in land, implements, &c.; while in Great Britain the outlay is 5*l.*, in Scotland 4*l.*, on an average of seven years. There is, however, in the same document another and a more pleasing aspect of these communities presented. "When I," says Sir John M'Neil, "visited the colony in 1853, the whole 3000 acres, except some patches of wood, peat moss,

---

* *Account of the Poor Colonies and Agricultural Workhouses of the Benevolent Society of Holland,* by a Member of the Highland Society of Scotland.

† *Tour in Holland, the Countries on the Rhine, and Belgium, in the Autumn of* 1838.

and the roads and canals, were in regular tillage, the 420 houses, then occupied by the colonists, were clean and comfortable; the colonists were well fed, well clothed, and healthy; the schools and workshops in active operation; and that great sheet of cultivation studded with its neat cottages, inhabited by numerous families possessing so much of material well-being, like an oasis in the desert of stunted heath by which it is surrounded, was certainly a most attractive spectacle. Had I prosecuted my inquiries no further, I should have left Fredericksoord with the conviction that the scheme of free colonies had been completely successful, and that 420 families which had been a burden upon the benevolence of the community, had been transformed into a prosperous colony of small farmers, enjoying the reward of their industry in an amount of comfort exceeding that of the majority of their class in the same country."*—p. 6. For our present purpose it is not necessary to prosecute the inquiry further than the point to which this eloquent peroration leads.

The paragraph quoted appears to justify the conclusion that in Holland numbers of paupers of wandering habits and a low moral type may be provided with houses at the rate of 10*l.* per individual, maintained at a loss of about 10s. per individual per annum, taking a family as consisting of four, in the agricultural colonies, and though failing to maintain themselves, as was anticipated, they succeed in reclaiming extensive wastes, in assuming a regular and respectable deportment, and in making the desert blossom like the rose.

The most recent intelligence on this matter is contained in Murray's *Handbook* for 1856, where, after employing the facts, even the phrases supplied by Sir John M'Neil, it is observed, "though the attempt has not realized the sanguine wishes of its proprietors, nor is likely to do so, yet it has succeeded in the benevolent object at which it aimed, by rescuing many hundred individuals and families, previously paupers and friendless, from vice and destitution, making them useful members of society, and in rendering fertile and profitable large tracts of land previously desert and useless."†

It may be instructive to bring together some of the peculiarities by which these destitute and dangerous classes are distinguished, and to which are attributed, in part, the failure of the scheme for their redemption, as well as the special arrangements to which they are subjected; as approximating them in various degrees and at different points to the insane, and to the system which must be pursued when colonies of the insane are founded. They are

* *Eighth Report of Board of Supervision for the Relief of the Poor.* Edinburgh, 1853.

† Murray's *Handbook for Holland, Belgium, Prussia, &c.* 1856.

described as inept for agricultural occupations, as having a want of economical habits, as belonging to a race or class which, by want of energy and economy, has been reduced to indigence. But this propension becomes a hereditary taint, for the children of these men, though educated and improved in habits, cannot be taught industry, the spirit to maintain themselves, or to trust to their own exertions for a livelihood. It is asserted that so deficient is physical energy and strength in the race, that fifteen colonists are required to perform the field work of one good day-labourer. Subjected to a most minute and rigid code of regulations affecting their labour, time, the property committed to them, cleanliness, their moral and religious responsibility, to the violation of each of which penalties are attached; and watched and warded without ceasing; in order to economize supervision, and to secure the effects of example and co-operation, they work in gangs. Evasions occur, and each colony is surrounded by a wall or ditch, and no inmate is allowed egress without a written permission. But should these free colonists become indolent, refractory, incorrigible, they are transferred to a gigantic seclusion-house, where escape is impossible, and where punishments of a graver kind are inflicted.

There is in all these communities, widely separated as they are in other respects, a provision for the preservation of the family tie, under grave restrictions, it is true, and of the sacredness of home, within a guild or brotherhood, for the union of freedom within a limited range, with constant superintendence; there is the mingling together of the governing and the governed, yet the subjection to supreme authority, and the extinction of all individuality of purpose. These results have followed the training and isolation of sane but not altogether healthy or sound minds. In certain of these products may be observed what it is desirable to secure in any modified disposal of the confined insane. For the grand object is not classification, is not to promote greater activity in employment, or more remunerative efforts; it is not to rule by division, although all such justifiable ends may incidentally arise; but it is to introduce a new element, a new form of solace, and support, and happiness into the treatment of the insane—one which was unavoidably excluded from large communities and hospitals, but which seems necessary to the health of the individual, which is sought both to intensify joy, and in seasons of sickness, and sorrow, and despair. It has been long known that consentaneous action, confederation, conspiracy are almost unknown among the insane; that the formation of friendships, that intercourse, are indications of returning health, that they are likewise the means of accelerating convalescence, and, where that cannot be aimed at, of imparting to chronic and continued disease many of the attributes of health. The groups, the cliques, formed in galleries, are suggestive of family life. Single rooms in asylums are not always places of seclusion for the noisy or contentious; but retreats, rewards for the reserved, the well-disposed, and which, under such management, so frequently assume the aspect of a private dwelling. In truth, the triumph of social reunions—of that part of moral management which brings different sexes, ranks, members labouring under different forms of alienation together for a common object, in one common pursuit or pastime, is but a step in the direction of establishing smaller communities, and of bringing each participator nearer into contact with the usages and feelings of his family and home.

> "The first sure symptom of a mind in health,
> Is rest at heart and pleasure felt at home."

It may be argued still further that chiefly where the internal arrangements of asylums most nearly approached the aspect and tone of domesticity, have they been most successful and beneficial in treatment; and that it has been the desire of all concerned in regulating the life of the docile and incurable insane to banish from their home equally the vastness of the American hotel; the iron bars, although they do not necessarily "a prison make;" the sickly sameness or the lugubrious paraphernalia of the infirmary, and the severe and parsimonious bareness of the workhouse; and by simple appliances, common furniture, and familiar objects, to reproduce what the mind has associated with its earlier and rational states, and with places and persons the recollection of which is calculated to withdraw it from its morbid and miserable self-analysis.

An asylum should assuredly neither be a prison nor a palace. Whether it contains one apartment or one hundred, a single inmate or a group, it should provide for safety without the iron aspect of security; and while affording that degree of comfort and those arrangements dictated by art which the mental condition of the patient demands, it should shut out splendour and ornamentation, which are incongruous to the object of an hospital or sanatorium, which may involve and conceal parsimony and error in other matters, and which, although remedies in certain cases, do not necessarily contribute to minister to the mind diseased. While space and air and light should be lavishly given, gargoyles and gilding, vestibules and grand staircases, may be doled out with a niggard hand. Nor is it absolutely necessary that a cottage should be Elizabethan, or lodge-like, or even in rigid keeping with the supposed picturesque, or rather that it should realize the ideas of the architect in the latter respect; as we know from high authority that with this quality much of the discomfort and squalor and sickness which are found in such residences may often be associated even in England. Nor is it proposed that such residences should come under the category of cottages ornées; a mansion house on a reduced scale, with books and busts and vases and verandas; lilliput libraries and embellishments *à la* Tom Thumb; but that they should possess, without the defects and vices, much of the homeliness of the houses to which the inmates were accustomed, and which they had learned to love and to use, and to which they must return. In fact, in order to secure perfect harmony between the early impressions of the inmates and their compulsory residence, it would be wise to exclude luxuries, and to introduce the time-honoured eight-day clock, the ambry, the wheel, the shelves of delf dishes, the domestic cat. Again, it would be absurd to convert these cottages into a means of training the occupants to the appreciation of a higher social condition, except in so far as the order and cleanliness, the mode of preparing the food, may incidentally lead to such a result, while it is securing, as it is intended to secure, the return of health.

In this inquiry there has been a tendency to confound the cottage system with that in which it is proposed to have a number of block-houses in place of one, an arrangement which it may either supplant, or be associated with. This division may be for the purpose of placing the sexes in distinct buildings, as in Pennsylvania; or for the segregation of the convalescent, as at Illenau; for the elimination of the agitated, as at Salpetrière; for the separation of the patrician and plebeian; or for association in pursuits, as is attractively shown in the advertisement of the Colonie de St. James; but whatever the motive, and however excellent the object, the arrangement is not that now proposed. It is a mere multiplication of asylums, and does not, and is not intended to secure that family life, that individualization, which is desired. These observations may be extended to the succursal dependencies in various institutions, to the pavillons at Vanvres, the chateaux at Brislington, the cottages at Aberdeen. They are detached portions of the asylum, but they do not differ from it; in regulations, regimen, furniture, they are identical. With signal advantages peculiar to themselves, they do not realise the ideas of the domestic circle, the relations of parent and child, guardian and ward, host and boarder —not even master and servant; nor of the frankpledge which has been attempted, and which might be so beneficially engrafted upon such communities. What, then, is a Cottage Asylum? Definition should be avoided as well as dogma; and even description should allow for amplification and development in the same direction. As at present contemplated, it is intended to convey the idea of an establishment in which, around or in connexion with a large hospital or sanatorium for certain classes of the insane, there shall be smaller buildings capable of containing families of which other classes shall form members; all members of the community being equally under the care and authority and constant supervision of a central medical staff. What is wanted is a village around a manor house. Vast parks and farms are not expected; and as the mere location in cottages will not increase the gross population of the asylum, the ordinary amount of ground, the ordinary proportion to each patient will suffice. But the inhabitants of this park hamlet must be entirely under moral control; an allegiance which can only be maintained by the limitation, not the abrogation of physical liberty, by sustained supervision, and the contact of a trained and trustworthy body of guardians.

The enormous outlay involved in the erection of modern asylums has suggested various economical expedients. It is natural that a maximum cost of 360*l.*, and a mean cost of 154*l.* for the accommodation of each patient, including land, should inspire alarm in the theorist, as well as in the ratepayer, and induce a reaction which has militated against the proper disposal of the insane. The effects have been even more disastrous than a mere preference for cheap structures and furnishings; as it has led to the adoption of a stereotyped form of building, recommended rather by the views of architects than by the experience of psychologists; and has afforded a pretext, in some parts of the country, for indefinitely delaying or declining the erection of Asylums at all. It is well ascertained that buildings possessing certain internal arrangements contribute greatly to promote the health and to facilitate the classification and the management of the inmates; but great differences of opinion still exist, and among the most competent judges, as to whether there be any one special arrangement deserving preference, or many, and some of these of the simplest character which is compatible with the objects in view. Nor is it yet demonstrated that the usefulness or manageability of a house is in proportion to the thousands lavished upon its ornamentation, nor even that the best plan may not be the cheapest. The famous Sonnenstein was anciently a castle where the redans and the redoubts were converted into day-rooms and dormitories, the glacis into gardens; if Bicêtre rose on the ruins of an Episcopal palace, Salpetrière is a modified manufactory; the scene of Jacobi's labours at Siegburg and of Esquirol's at Charenton was a convent; and the most highly decorated asylum I have ever seen was a half-ruined resort for pilgrims in Bruges.

Although, perhaps, in Asylums, as in Governments, the best administered is best; an able executive sometimes bringing out splendid results from the most clumsy and unmalleable materials; an able and active and inventive mind compensating for the defects and inertia of stone and lime; yet it is of importance that improvements should be introduced into such structures, that the requirements of science should be made compatible with as small an original expenditure as possible; reserving all extreme liberality for the treatment rather than the mere lodgement of the insane; but, leaving the discussion of the comparative merits of linear, or H plans, and of one or many distinct blockhouses to others, I would venture to take advantage of this difference of opinion, of this state of incubation or transition, in order to point to the erection of central houses of somewhat less gigantic dimensions, in conjunction with cottages, as one means by which the difficulty might be overcome and the expense avoided. Considering the simplicity of such a measure, and the ease with which it can be carried out, it being a provision which affords unlimited scope for addition and for gradual enlargement: and the fact that a large majority of the pauper and poor insane, in Scotland, at least, have resided, and do actually reside, in huts and hovels, and under circumstances more adverse than can be conceived to continue in any public institution; it is curious that no such plan has yet been tried nor suggested. The houses which have hitherto served as asylums, or places of detention, for one or two cases, are of the most primitive description. It is doubtful whether any innovation has crept in since the Roman invasion; and that then the type had been borrowed from other and earlier races; only that the plagiarism could not be detected in the perishable materials of which such habitations were formed. And yet these fragile tenements remain to tell their tale longer than could have been conceived. Esquimaux huts last, perhaps by the aid of congelation, for two hundred years, and mud-houses closely resembling those still found in Cambridgeshire are met with in South America three hundred years old. We have little concern with the fabrics, fondly named camps and castles, which have left nothing more than a faint outline of greensward on the hill-side, or with the ashes, and bones, and querns, which reveal a glimpse of their internal polity; but when, near some sepulchral cairn, are disclosed huge masses of granite thirty feet long and eight feet wide, the rough side out, and where these walls converge to the top as a substitute for an arch; or where we encounter on the slope of a glen, by the margin of a brook, beneath eight or ten feet of peat moss, oval passages of stone, about six feet in diameter, surrounded with the remains of pointed hazel stakes, or poles, the beams with which the walls were framed, we recognise the winter and summer dwellings, sometimes the villages of the true Caledonian peasant and poor of Dalreadic times, possessed at that time, perhaps, by the same class throughout Europe, and closely resembling some of those still occupied in remote districts and containing the insane.

Nor must these subterranean holes and wigwams, "that mole and rabbit mode of society" as it has been designated, be complacently carried back to primeval savagedom, when insanity was not; for Sir Richard Colt Hoare, in his *Ancient Wiltshire*, says, "We have undoubted proofs, from history and from existing remains, that the earlier habitations were pits or slight elevations in the ground, covered and protected from the inclemency of the weather by boughs of trees and sods of turf."* Thomas Kirke, a Yorkshire squire, writing in 1679, when there was no Bedlam in Scotland, describes the gentlemen's houses "as generally of a fortified character, with strong iron grates before the windows, &c. The houses of the commonalty are very mean, mud wall and thatch the best. But the poorer sort live in such miserable huts as eye never beheld; men and women pig together in a poor mouse-hole of mud, heath, and such-like matter."† "Old men remember," writes Mr. Cosmo Innes, "when the dwelling of the Scotch peasant was not secure against wind or rain; with no window, or none made to open, with damp earth for floor, with dunghill and green pestilent pool at the door. The 'black hut' that is still to be seen in a few glens of the Highlands, is a less unhealthy abode than the houses of the yeomanry and peasantry of three-fourths of Scotland were half a century ago."‡ It requires no stretch of memory nor of fancy to realize this description. Such habitations still remain in thousands: they are accessible to the curious in mediæval architecture who yearly tread Iona and the West Coast; and they have been recently introduced to public notice in an authoritative document, and expressly on the ground that they are the "Cottage asylums" for large numbers of the insane poor.

"The Shetland cottage is usually built of undressed stone, with a cement of clay or turf. Over the rafters is laid a covering of turf, or sward, and above this again is a thatch of straw, bound down with ropes of heather, weighted at the end with stones, as a provision against the high winds which are so prevalent. Chimneys and windows are rarely seen. One or more large holes in the roof permit the escape of smoke, and at the same time admit light. Open doors, the thatched roof, and loose joinings, everywhere insure a certain ventilation."§ "Passing through the byre, the human habitation is reached." "Part of the house is used in winter as a privy."|| "There is a barrenness and desolation about the misery of a Harris house that is tenfold more depressing. It is a poor house and an empty one; a decaying,

---

* Quoted in Wilson's *Archæology and Prehistoric Annals of Scotland.* 1857.

† Chambers' *Domestic Annals of Scotland,* vol. ii. p. 407.

‡ *Scotland in the Middle Ages,* p. 318.

§ *Second Annual Report, Board of Lunacy, Scotland,* p. 214. 1860.

|| Ibid.

mouldy shell, without the pretence of a kernel."* "The typical Lewis house is not simply a long unbroken range. It rather consists of a major block, of forty or fifty feet, with a small porch-like wing at one end in front, and a larger projection or attachment towards the other end behind, which last serves as a barn. Access to all is gained by one door. By this you enter the so-called porch, and on one hand you find that which is now seen in most antiquarian museums—the quern, not kept there as a curiosity, but as a regular fixture, and a thing for daily use. Opposite this is the stall for lambs and calves. As you pass from the porch to the major block, you first encounter the byre, and in summer, after the planting of the crops and the removal of the annual accumulation, you here descend a step: in early spring, however, you ascend. The cattle rarely leave the house during winter. On one side of the fireplace, supported on two pieces of turf or on two large stones, is a plank, which is the seat of the men of the household. On the other side is a rough three-legged stool for the wife. All sexes sleep together in the beds. The house itself is constructed of rough, unhewn stones; walls five or six feet thick, with an outer and inner facing of dry stonework, the intervening space being turf. The rafters do not overlap the wall, but terminate upon its inner edge, so that the rain falls from the roof into and not over the wall, which is therefore always damp. Walls six feet in height, and the door can only be entered by stooping. On the top of the wall, round the roof, there is often a footpath, on which children, sheep, dogs may be constantly seen. In one case, the public footpath to a neighbouring township of crofters led me over the end of one of these houses. There is no window; yet when the spirit of cleanliness, order, and industry enters it, comfort is then found where comfort seemed impossible."

"It has been thought best to divide the habitations of the lunatics visited into two classes—the Saxon and the Celtic. The first is intended to include dwellings built of stone and lime, roofed with slates, floored with wood or pavement, having glazed windows, a chimney, and a fireplace, being generally divided into a 'but and a ben,' and sometimes having a garret or garrets between the ceiling and the roof. Of these there were thirty-nine entered. The second comprehends dwellings constructed of stone and mud, or more generally of sod; thatched with fern, or straw and mud, or covered with turf; having earthen floors, or these only partially paved with stones from the shore or the burn; where there are no chimneys; where the fire is in the centre of the single apartment which rises to the roof-tree, or is placed at the gable, and contained in or on a few stones, and where there are

* *Second Annual Report, Board of Lunacy, Scotland,* p. 214. 1860.

rarely glazed windows. Of these, constituting the lowest order of the black houses, costing about 8*l.*, 120 were entered. There are, however, hybrids between these classes, created generally by the introduction of some Saxon novelty, such as a grate or a chimney; and there are places and parishes where these rude arrangements are made consistent with a certain amount of cleanliness and comfort." "Although the following description applies to a Celtic house, it is not intended to serve as an example of the class, but as illustrative of dwellings provided for the insane poor, and in which they must continue to reside until they are removed to an hospital, or until the existence of such an establishment, and such high-class accommodation as it will afford, will suggest or compel a corresponding improvement in the circumstances of those who are allowed to remain at home." D. K., an idiot, with father, who is old and infirm, and a pauper. House a small hovel, built of moss sods cut from its site, or the surrounding almost unapproachable bog. The turf is built around a framework of hazel poles. Floor of spongy moss, except where some rough stones have been placed by occupant, who of course sits rent free in this ditch. All around is an unsafe quagmire, baked hard, it is alleged, by the sun; but during nine months soft and spongy, the position being selected in order to avoid expense. Four similar huts are close at hand, all inhabited by paupers, one of which, at least, was more wretched than that of K. Even were these cabins habitable, it is monstrous to place them where they are. K. has lived in his hut, or one on the same spot, for seven years. The interior is dark, damp, dirty; so small, that the reporter had difficulty in standing upright; so rude, as to remind him of the wigwam of the North American Indian.* A somewhat analogous but superior structure is seen in Cambridgeshire. "After a labourer has dug a sufficient quantity of clay for his purpose, he works it up with straw; he is then provided with a frame of eighteen inches in length, six deep, and from nine to twelve inches in diameter. In this frame he forms his lumps in the same manner that a brickmaker forms his bricks; they are then packed up to dry by the weather; this done, they are fit for use as a substitute for bricks. On laying the foundation of a cottage, a few layers of bricks are necessary to prevent the lumps from contracting damp from the earth. The fireplace is lined, and the oven built with bricks. I have known cottagers, where they could get a grant of land, erect a cottage of this description at a cost of from 15*l.* to 30*l.* I examined one containing two good lower rooms and a chamber, and which was neatly thatched with straw. It was a warm, firm, comfortable building, and might last for centuries."†

* *Third Annual Report, Board of Lunacy of Scotland.* 1861, *passim.*

† Denson's *Peasant's Voice*, p. 31, quoted pp. 178, 179. *A Practical Work on the Management of Small Farms,* by Feargus O'Connor, Esq. 1848.

These Celtic huts are open to condemnation for many reasons: but while some of these apply to the form, a far larger proportion are directed against the squalor, the misery, the discomfort which originate in the habits and tendencies of the race by which they are possessed. A Highland proprietor has offered to the writer as an explanation of the wretchedness of these places, that their demolition is determined upon, that the erection of such is discouraged, and a tacit premium set upon their extinction. But, forty years since, the superior many-roomed homesteads of this class were neither despised nor doomed; they were of larger size, they sheltered a more affluent and educated people, and gave tokens of tidiness, and taste, and elegance which have passed away. It is not gravely proposed to surround Asylums with such structures, with the genuine "Feigh Dhu;" but cottages such as experience has brought to be typical of the district, may be found to unite the requirements of seemliness and suitableness; and it is highly probable that in Highland scenes, where some of the projected Asylums must be located, a building of simple form, bold proportions, and rude materials, may be better in harmony with surrounding objects than those trim, painted toy-boxes which Walpole loved, Shenstone sung, and George Robins would have delighted to advertise. It may, however, furnish data as to the cost of huts in which lunatics actually live, and enlarge our views of the whole subject to state, on the authority of Dr. Howie, Ardnamurchan, that the lowest type described may be erected for 30*s*., or, if two apartments are provided, for 2*l*. or 4*l*., according to the distance from which the turf may be cast and carried. In other parts of Argyllshire, where the walls are formed of stone without mortar, there is an outlay of 8*l*. 10*s*.; and, in order to show the practicability of such a feat, the estimate of a local architect is given, who, even in the decline and fall of those edifices, has built many.

| | £ | *s*. | *d*. |
|---|---|---|---|
| "For building | 4 | 0 | 0 |
| Eight dozen cabers, at 1*s*. 6*d*. | 0 | 12 | 0 |
| Thatching | 0 | 10 | 0 |
| Two windows | 0 | 10 | 0 |
| Two couples | 0 | 10 | 0 |
| Four joists | 0 | 4 | 0 |
| Four dozen cabers | 0 | 6 | 0 |
| Ropes | 0 | 2 | 0 |
| Composition for floor | 0 | 5 | 0 |
| Two doors, 5*s*. each | 0 | 10 | 0 |
| Four days cart and horse | 1 | 0 | 0 |
| Total | £8 | 9 | 0" |

In the north of Inverness-shire the expense would amount to

10*l.*; and in the same district cottages of stone, pointed outside, flagged, provided with glass windows, with an open interior roof, and thatched with straw, cost from 20*l.* to 50*l.*, while in the central parishes of the same county, on a turf-dwelling of two apartments, and without window or chimney, 13*l.* will be expended; and on one of a superior kind, possessing these advantages, and a closet between the lateral rooms, 28*l.* All testimony goes to show that the time which such cots remain habitable depends, in great measure, upon the manner in which they are kept; that the presence of a chimney, flooring, and windows, place the pointed stone house nearly upon a par with the built stone house, and render it compatible with great cleanliness and neatness.

If, by a long stride, we pass to the town residence of the swart smith or miner in mineral districts, as black and cheerless as the shielings we have left upon the mountain, in dwellings of two rooms, measuring 16 feet by 11, exclusive of the two beds in each, which implies that they are intended to contain at least eight persons, we find that the mason work amounts to 37*l.*, the wright work to 34*l.*, plaster work, 4*l.* 10*s.*, and plumbering and slate work, 10*l.* 10*s.*—in all to 86*l.*, or 10*l.* 15*s.* per head; or, where they assume the name and capacity of double houses, and are supposed to be adapted to the reception of twelve persons, the gross outlay is 145*l.*, or 12*l.* per head. If we continue our search in Aberdeenshire, a most intelligent correspondent states that crofters, when they build for themselves, by dispensing with the loft or garret, as they involve joists and flooring, and by dint of great economy, get up a house, not counting their own labour, for about 30*l.* or 40*l.*; or, where an upper story or loft, a most essential element in our estimation, is added, from 60*l.* to 80*l.* This dwelling may easily, and comfortably, and decently contain five individuals; or, should we pass into the adjoining county of Elgin, another authority affirms that a cottage, thirty-six feet over walls, consisting of a good "but and ben," flagged and floored, with garret or loft, and thatched with straw, would cost from 42*l.* to 45*l.* The walls inside would have one coat of plaster on the wall, no lath being used. The partition between the kitchen and room would get two coats of plaster. If the cottage were forty-two feet over walls, consisting of a kitchen, a closet, and room with a garret, flagged, floored, and thatched with straw, the cost would be from 45*l.* to 50*l.* If guided by our M'Cullochs and landscape painters, a thatched cottage forms an essential part of the picturesque. It is a "thing of beauty," though not "a joy for ever," as in some localities, and even when the material is pulled brechan, or fern-stalks, or heather, it requires renewal every seven years.

When we find that, even in England, in the fourteenth century, gentlemen's houses were regarded as extraordinarily well provided for if containing three or four beds; that in Elizabeth's time cottages consisted only of a single room, and that chimneys, a discovery of which it is said Vitruvius had not a glimpse, were then introduced, we do not recoil so much from the present state of the dwellings of the poor on the extreme verge of our island.* Of the gradual progress of the English cottage to its present unsatisfactory state, little has been recorded. "Of the great body of the people, of those who held the plough, who tended the oxen, who toiled at the looms of Norwich, and squared the Portland stone for St. Paul's, very much cannot be said; history was too much occupied with courts and camps to spare a line for the hut of the peasant, or for the garret of the mechanic."† Nor are we surprised to discover that a century ago the mud-huts in Bedfordshire, like all others of their class, were huddled together, dirty, ill built, ill drained, imperfectly lighted and watered, and altogether so badly conditioned and unhealthy, as to be unfit for the residence of human beings. It is pleasing that the movement for the improvement of these houses of the labourer, which is about to culminate in a legislative enactment, began on this spot, and, by the direct act and exertions of the philanthropist, the prison-cleaner, John Howard.

In Devonshire stone cottages, containing two public rooms, 12 × 11 and 12 × 8, and three bedrooms above, can be built for about 60*l.* The cob-house would cost somewhat less. From another trustworthy source we are assured that the wages of a farm labourer will seldom fairly admit of his paying a rent of more than 1*s.* per week, besides the value of the garden ground, if any. The lowest cost of building even a good four-roomed cottage is from 70*l.* to 80*l.*, according to the material; and the cheaper the building, of course more expensive and earlier repairs are involved. There is reason to fear, however, that such houses are of an inferior character as to internal arrangements, and that the cost is under-estimated; as Sir L. Palk, in moving the second reading of the Labourers' Cottages Bill, detailed cases of gross immorality resulting from overcrowding, which imperatively called for some legislative check. The object of this measure is to enable owners of estates to raise money, not exceeding 140*l.* per cottage, for the improvement of the cottages of labourers by a

* Hallam's *View of the State of Europe in the Middle Ages*, vol. iii.

† *The Dwellings of the Labouring Classes, their Arrangements and Construction. Illustrated by Reference to the Model Houses of the Society for Improving the Condition of the Labouring Classes.—The Model Cottages built by Windsor Royal Society*, by Henry Roberts, F.S.A., &c. Third Edition, 1853.—Dr. Watson Wemyss. *The Economic and Sanitary Improvement of Dwelling Houses for Agricultural Labourers.* 1860.

first charge on land.* We have not means of knowing the kind of dwelling which is proposed, nor whether the amount to which the charge is to be restricted may not be intended, and the intention is natural and justifiable, to render the building ornamental, or more in keeping with the domain, or the architecture of the mansion-house, of which it may be said to form a part. The originators and directors of so laudable an enterprise must be familiar with the following words:—"Fitness of style, justness of proportion, and internal comfort, are perfectly consistent with picturesque effect, and with that spirit of strict economy which is indispensable in cottage architecture for the masses of the people."†

In this country, however, many examples of cottages of a very superior description, and very similar to those which may be placed in connexion with asylums, and erected at a very moderate cost, exist. The following extract is from a newspaper in which this and congeneric topics have obtained a conspicuous and most useful attention:—

"In one block of cottages that we visited we found no less than five separate apartments—a kitchen with asphalte floor, a parlour boarded, a bed-room off the kitchen, with two beds, intended for the parents and the young children, and two small bedrooms off the parlour (each half the size of the one off the kitchen) for the use of the elder boys and girls. All the bedrooms are well lighted and ventilated—not only by means of the windows, which are so arranged in conjunction with the windows of the kitchen and parlour, that a fine draught of fresh air passes through them whenever the windows are put down, but also by ventilators in the ceilings connected with the outer air by a pipe passing up along the outside of the vents. Each of the cottages has a back door, more than half of which is of glass. This serves to light a commodious press and a scullery at the end of the passage. The bed-rooms have all fixed bedsteads, the kitchen and the parlour are provided with neat grates, and there are handy presses in both, with additional shelving for dishes in the kitchen. The kitchen and parlour are each about thirteen feet by twelve feet; each cottage measures thirty feet by twenty feet over all. The cottages are surrounded with a 'rone' to receive the rain water, which is collected in two large barrels, the site is well drained, each cottage has its necessary at the back, and, besides a garden behind, there is a neat little plot for flowers in front. The total cost of each cottage is stated to have been about 100*l*. Such cottages are of course uncommon, and many farmers declare, and facts bear them out in not a few instances, that ploughmen do not care to occupy more than one room. In the case of the cottages we have alluded to, however, the occupants appear fully to appreciate this excellence."‡

---

* *Times*, May 9, 1861.

† *Letter, Duke of Bedford to Earl of Chichester. Dwellings of Labouring Classes, &c. Windsor Royal Society.*

‡ *Scottish Farmer*, April, 1861.

Amid hundreds of others, I have visited and examined several of these in different localities, and with a special reference to the present inquiry; but in conveying some idea of their aspect, construction, and expense, I shall adhere literally to works in which they have been described by the proprietors or their representatives, and to whose philanthropy they are due. It is pertinent to these observations that in some of these groups were seen well-kept and happy pauper lunatics.

A square of houses was erected several years ago under the auspices of the Marquis of Breadalbane, on his property at Acharn on Loch Tay. All these are built of stone and slated, and obtained the unqualified approval of the Highland and Agricultural Society of Scotland as regards situation, drainage, substantiality of structure, internal accommodation, and outward appearance, and as, in every instance, showing the very marked improvement in the condition of the people induced by these commodious arrangements. The attractive exterior of these cottages might to many prove an objection. In one set there is one apartment on the ground-floor, measuring fifteen feet by ten feet, and two on the second, reached by a staircase, measuring respectively fifteen feet by eleven feet and fifteen feet by ten feet. In another set the arrangement is reversed, there being two rooms on the ground and one on the upper floor, the dimensions corresponding to those already given. These houses being built in blocks and of stories, secure a considerable saving in gables, roofing, &c. The entrances, however, and other arrangements, render them entirely distinct and private. The average cost appears to have been 65*l.* The total expense, including charge for excavations, pavements, zinc windows, grates, common to all, was 986*l.* 19*s.* 7*d.*, but deducting from this contracts for offices, the outlay amounted to 844*l.* 5*s.* 5*d.*, which divided over fifteen cottages, gives the average stated above, 65*l.* 16*s.*; but by dividing the cost of the offices and the contingencies over the whole equally, we have, for out-of-door offices, about 17*l.* 4*s.* to each cottage, and this added to the cost of one cottage of each class, gives a total of 56*l.* 8*s.* for each cottage of two apartments, or, taking the family at four individuals, though there were actually a larger number of inmates, 14*l.* 2*s.* per individual, and of 86*l.* for those of three apartments and offices, or 21*l.* 10*s.* per individual. It will be obvious, however, that it is for the price of such cottages *exclusive* of the outlay for offices that we have to deal.

About thirty years ago, J. J. Hope Johnstone, Esq., of Annandale, M.P. for Dumfriesshire, adopted the plan of granting to cotters, tradesmen, and hired servants of good conduct, a lease of twenty-one years of the house-stead and large garden at a rent of 5*l.* The tenant erects the house at his own expense, excepting the price of timber, which is given from the estate, and hewn freestone for chimney heads, door and window rybots, jambs, &c., in all costing the proprietor from 5*l.* to 6*l.* The cost in 1844 was—

| | £ | s. | d. |
|---|---|---|---|
| Welsh slates . . . . . . . . . . . | £4 | 10 | 0 |
| Mason and slater . . . . . . . . | 5 | 10 | 0 |
| Joiner, and windows, two in front and one or two in gables . . . . . . . | 7 | 0 | 0 |
| Price of lime, and other outlays . . . | 4 | 0 | 0 |
| | £21 | 0 | 0 |

In 1859, from the higher rate of tradesmen's wages and better finishing, this cost may have increased to 30*l.* or 35*l.* Any additional cost is confined to the tenant's own work, in raising and finding stones, assisting masons and carriages which, in most cases, they get in kindly exchange for farm work to farmers and neighbours. I have lately seen in two asylums mason and carpenter work to a considerable extent executed by patients and in a creditable manner, so that a slight saving might be expected in the erection of cottage asylums, while employment of a novel description would be afforded to those who might benefit by their own handiwork.

The smallest house is generally thirty feet long by nineteen feet wide over walls. Those most recently erected are higher, the under floors and lofts are boarded, the room-end occasionally stoothed and papered. It must be admitted, however, there is still too much crowding, with less ventilation and fewer conveniences than are desirable. The early drafts upon the family by the departure of children into service; and the invariable practice of leaving the doors open, and in a manner living in the open air, in part compensates for these disadvantages: but the radical remedy would consist in restricting the number of inhabitants. These houses, about seventy in number, are in general placed in single dwellings along twenty miles of turnpike or parish roads, whitewashed, comfortable, and clean in appearance. Many of the original leases have now expired, but few changes have occurred. The results have been most satisfactory, as might have been expected from the security of tenure, the regard had to the respectability of the leaseholder, and the constant supply of labour.

The aspect and situation of these dwellings differs so much as to diversify and beautify the country. The interior is likewise variously arranged, the number of rooms corresponding (within certain limits) to the size and wants of the family; and their position, although a general principle may be seen to run through the whole, being regulated by other objects. The first of about twenty entered was occupied by nine individuals. In some respects it realized that architectural *summum bonum,* a rectangular building; contained three rooms, two of which measured thirteen feet by thirteen, feet and eight feet in height, having fireplaces; and garrets of good size and provided with skylights, which were unoccupied, but might have accommodated several additional inmates. The second, where the family consisted of six and a servant, had three rooms, a shop, and offices; the passages were paved, the rooms boarded. In the public department there were two windows, in the others one. The form in this instance was that of a main block with receding wings. The third, and this presented the most common type, had two apartments and a closet behind communicating with the kitchen; and lofts, which were not, however, converted to any use. In this the anathematized box beds had disappeared, and recesses substituted, built in the wall, which was plastered over stone and lime. Considerable improvement had been recently introduced into this cottage; the original floor of clay and rough stones had given place to planking, the walls were papered, and by the aid of a blooming garden in front, and rigid cleanliness and tasteful contrivances within, the observer was led away from brick and mortar to romance. But in the next, built about twenty-six years ago, which was complained of as cold and damp, and stood as it had been placed, embowered in trees, with an uneven pavement of stones and flags, and a roof of the same material, there were found three rooms, two with fireplaces; the lofts being convertible into garrets, but of difficult access; the whole capable, the occupant was confident, of receiving eight or nine inmates, and undoubtedly of separating or properly dividing five or six. Were it obligatory to transplant Gheel to this country, it should be placed in Annandale, where plain but comfortable houses, capable assuredly of improvement; where substantial prosperity, a smiling and apparently happy valley, and a respectable if not a pattern peasantry, offer greater encouragements for such an experiment than can easily be met combined elsewhere.

I am prepared, but not disposed, to append either plans or precepts for guidance in the modification of such dwellings as have been adverted to, or in the construction of succursal cottages upon a more suitable principle. There is nothing complicated, nor recondite, nor special required. If plans either in verification of what has been advanced, or in furtherance of the project advocated are sought for, they will be found in the *Model Cottages built by the Royal Windsor Society,* the *Transactions of the Highland and Agricultural Society of Scotland, Annual Reports of Association for promoting Improvements in the Dwellings, &c. of Agricultural Labourers in Scotland,* &c., furnished for a different but equally laudable purpose; drawn by architects of eminence, sanctioned by practical men, and recommended upon moral as well as upon economical grounds, and, in one instance, after having been tested by occupation for a quarter of a century. If directions were craved they might be supplied from the noble words of the present Prime Minister of England, who, after proclaiming that proprietors and tenants in affording accommodation for the agricultural labourers must not look too much to receiving a per-centage in the shape of rent; that it was only by promoting the comfort, health, and decency of this class that they could obtain the full value of agricultural possessions, added, "these cottages should have at least three sleeping rooms, one for the heads of the family, one for the males, and one for the females," and he might have safely added a window and a fireplace in each room. Dr. Conolly, in commenting upon the objections to the expense of county asylums, said long since, but for all time, that the benevolent consideration which is so cheerfully given in all Christian countries to the sick poor who are not insane, ought, at least, to be as freely extended to those labouring under sickness and infirmity of mind;* and it should be accepted as a corollary from this sentiment, that nothing sordid, nor paltry, nor penurious should, upon the mere score of saving, enter into the contemplated arrangements. But simplicity and order are in themselves beautiful. If anything be lost in decoration, there will be unequivocal gain in greater freedom, in the liberty of privacy, in living in their own house or room, fresh air, and sunshine. The central infirmary should, in relation to its special purposes, be superior and more commodious than ordinary dwellings; but it is conceived that some similarity should exist between the cottage within the precincts of the asylum and those without; and these brief notices have been accumulated to show how small the additions required in order greatly to surpass the original home, in order to accomplish the object in view; to explain how simple the elements are which we are called upon to work into a suitable form; and to contrast the circumstances in which the lunatics have lived, with those to which they must be transferred.

But connexion with such cottages does not alienate the lodger from the house of reunion. His privileges, many of his pursuits, his amusements, his meals will be accessible only under the spacious domes and arcades, if such there be, which shelter other classes of the community; while, superadded to these, are the quiet domestic life and engagements which are secured in his special dwelling. The opinion that family life may be created amid the crowds in a public asylum has been adventured by a French psychologist; but apparently in ignorance of what such

---

* *Construction of Asylums.*

communion imports; or, at all events, of what it means in this country, and of what is practicable in our asylums. If cottages, however, of the kind described, can be provided for labourers and artisans, the members of whose families are, or may be, numerous, and of different ages, where the arrangements must necessarily be complicated, it is obvious that the simplicity of the relation between one or two or three females and a mistress, or dame or associate, or between the same number of males and an attendant and his wife, will render the provisions for sleeping accommodation easy, especially as during sickness an immediate removal will take place; will place a large amount of space at the disposal of the guardians, and reduce the cost of accommodation per head nearly as low as if the patients were located in a turf or mud-house. In so far as this latter object can be effected, without infringing upon individual treatment and enjoyments, in fact, upon the rights of the inmates, the sphere of usefulness will be widened by affording opportunities for increasing the numbers of the general population.

I am not engaged in solving the problem of providing cheap accommodation for the insane. I think, however, that a conceivable argument against cottage provision is removed by demonstrating that dwellings have been erected and long used for sums of 60*l.* and 35*l.* These houses were intended for the class from which the inmates of asylums for the indigent may be expected to be supplied. They contain two or more rooms, sufficiently spacious for a family of six persons; of a construction susceptible of being heated, ventilated, kept clean; of having Pompeian hollow bricks in the walls, of being laid with tile pipes and broken stones and drains, and in which the inhabitants have possessed an average amount of bodily health and vigour. If in the smaller one, and in the larger of these permanent homes two rooms were set apart as dormitories, or if the patients passed the night in the same chamber with their attendant, a practice not inconsistent with the established customs of this country nor with those prevalent in all asylums, they would be lodged at a cost as yet unprecedented and even undreamed of by the most sanguine speculators. It does not appear that any peculiarity should be introduced into cottages, built according to sound principles and found to be comfortable. Any specialty would make them unhomelike. The class selected for incorporation with the attendants or dependants and their families would be to a certain extent trustworthy; the sense of responsibility would grow by being trusted; personal supervision and the parole of all for the integrity of each must compensate for defective honour and prudence, so that additional means of security may reasonably be dispensed with. But should it be deemed beneficial to add to the size of these dwellings, or to render them more ornate, more pleasing to the eye, though perhaps less like a cottage, these objects can be secured by a comparatively small additional expenditure; and even were the outlay doubled it would fall short of what has hitherto been entailed in providing accommodation for the insane. Objections exist to any great enlargement for the purpose of assembling together numbers of the insane, and the chief of these is, that it is fatal to the principle of domesticity and privacy which should underlie the whole enterprise. Nothing should dissever the tie between the recluse and the great body of which he forms a part, between the patient and the physician, and the means he employs to regulate the health, but the tie may be loosened and lengthened, or supplied by higher motives; but if these houses become merely retiring and sleeping places for companies of eight or ten, such as are associated under ordinary circumstances, they may be commendable on the ground of easy ventilation and quiet, but, morally, they will differ very little from the small and vicious, and vicious because small, dormitories in a block-house.

But there are other considerations worthy of note. In the manner proposed, ten, twenty, an indefinite number of patients might be withdrawn from the crowded galleries, from the Maelstrom of turbulent passions. This is not merely a gain of space. It is a gain in three ways; by relieving from the pernicious effects arising from the close and constant contact of unhealthy and therefore antagonistic natures; by facilitating management; and by protecting sensitive, and impressionable, and excitable minds from the influence of the more energetic. It is not proposed that such separation should be general, as the converse of some of these propositions may in some cases be urged, for if the calm become excitable by associating with the excitable, the excitable become calm by mingling with the calm; but within limits, the opportunity of even temporarily relieving the pressure which must, less or more, obtain in all large establishments by the removal of certain classes to separate buildings would be invaluable. It would, among other effects, greatly diminish the necessity for seclusion of the violent, which is resorted to nearly as often for the interest of those around as for their own. There would be provided an object of ambition to be sought, striven, lived for It is the claim and cry of many an inmate of a refractory ward, and it is hard to reject the petitioner, not for freedom, but to be removed from noisy, noisome, profane, dangerous companions, to some less frightful, some tranquil gallery. It is as often the craving of the calm and chronic for the society of the sane, for one breath of the "free, fresh breeze on mountain playing," for a brief indulgence of the sense of emancipation. It may appear overstraining the argument to pursue the benefit of the cottage system to its effects upon those for whom it is not designed, or to affirm that such a system would reach every inmate cognisant of its existence; but that the recognition of being almost, if not altogether, unworthy of bonds, involved in the act of transference from seclusion to a home, must operate curatively and beneficially upon all to whom it may ultimately apply, does not admit of question.

The distribution of such houses is not immaterial. It is not necessary that they should be marshalled into a parallelogram like a Spanish reduction, nor planted at measured distances along avenues like a Dutch colony; nor be isolated and surrounded with means of coercion or protection like a military settlement; yet regularity in arrangement, facility of supervision, and security, should all be cared for. As, however, two or more of them will serve as entrance lodges; as others may be the residences of gardeners, joiners, storekeepers, the position of a number must be determined by convenience, and of others by the nature of the site and by the extent of the grounds or farm, and, necessarily, by the conceptions of fitness entertained by Boards of Direction and builders. Such dwellings may be regarded as paying rent. If a celibate or a married couple, who it would be reasonable, or as reasonable as in similar combinations, to demand should be childless, be entrusted with such a section, it is fair that the extra duty, for such it can be held to be, should be compensated for by the superior kind of accommodation afforded; while the provision of a house at all must form an important part of the wages agreed upon. Although it may be legitimately objected to the presence of children, that they will fill up and exhaust the affections of the guardians and dwarf their exertions, to exclude them in all cases would be to monasticise and chill the group. I have entrusted children, and my own, to almost all classes of the insane, as a remedy and consolation, and have seen lunatics nursing infants who had the strange fate of being born in a padded room; and far from prohibiting, would prescribe the widow with children, or the unbroken family, as a nucleus for a certain number of cases.

By the respectable class of attendants such an arrangement would be regarded as an inestimable boon, and in the case of all others the withdrawal of temptation, the check, the control, the moral influence exercised by residence within the precincts, would repay a hundred-fold even a lavish outlay, were such necessary; for every superintendent knows that the liberty day, the liberty hour, the temporary absence, the evening visit to town, or family, are fraught with dangers and difficulties to both sexes, which rarely present themselves under discipline, and which recoil upon the happiness and reputation of the body to which the offenders are attached. The existence of such homes, and the advantages which they secure, would serve as an inducement to a superior class of candidates; and have been suggested and in some instances supplied with this object, where the more direct interests of the patients were not considered. The expediency of multiplying such guards and guarantees for human virtue is supported by the fact that from the ordinary constituents of the population there can be selected, after repeated trials, competent custodiers is not so certain as is supposed. Various tests of eligibility have been proposed and applied—education, particular professions, temperance or abstinence pledges, marriage, membership with the church to which the applicants belong, but all these have proved abortive or inadequate. It is not merely with the antecedents, it is with the capacities of the candidate that we have to deal. So naturally does the suspicion arise that deterioration is induced by long-continued exposure to contact with unhealthy minds, that two learned friends, Brierre de Boismont in 1854, and Dr. Sibbald during the present year, have instituted an inquiry into the ratio of mental diseases to the indigenous population of Gheel. It is certainly something to be bullet-proof. It is not enough, however, in such a grave matter, to prove, had success attended the attempt, that these Gheeloises were not more affected with insanity than their compatriots, who are merely clod compellers. There may be a worse effect than madness produced in the guardians of the insane by the performance of their duties. They may become irritable, hebete, callous, criminal. The notion that attendants become ill from infection, or imitation, or sympathy, is all but exploded; but the less extravagant insinuation that, apart from the effects of age and the tear and wear of anxiety, the mental and moral powers are taxed and ultimately suffer in this department of medicine, few will be found to doubt. But the thesis as to the Belgian peasantry is that they become *better*, that their habits of thought, almost their training and experience, are *transmissible*. But on looking at the soil from which these results spring, there are no reasons to discourage us in our present enterprise, as it appears that the means of education are nearly the same in both countries;* and that "il se trouve que la loi du développement du penchant au crime est la même pour la France, pour la Belgique, pour la Grand Duché de Bade, et pour l'Angleterre, les seuls pays dont les observations soient bien connu."†

---

* *Ducpetiaux on Penitentiary Reform*, p. 80, vol. iii. 1838. *Quarterly Journal of the Statistical Society of London.*

† *Sur la Statistique morale, et les principes qui soient en former la base. Par Ad. Quetelet.* Dec. 1846. *Mem. de l'Académie Royale de Belgique.* xxi., p. 99. 1848.

If individuals or small groups—and they should be as small as possible—join their associates at the public dinner, for the influence of society must not be superseded by the claims of segregation, and be accompanied by their matron or guardian, the kitchen arrangements and duties become occasional and slight; and if the industrial part of the population should be transferred to the single houses, and continued their trades in workshops, or in the fields, their homes would more and more resemble that of the husbandman, which is deserted, ventilated, cleaned, and set in order during the day, and occupied, lighted, heated, attractive before and after the hours of labour and activity. Or, should the aged and infirm, the dreamy monomaniac or the hopeful convalescent, prefer the quiet or busy circle round the kitchen fire to the large assemblies in the central house, there seems to be no good reason for disturbing what is a legitimate and pleasing part of the peasant's family life. The experience of attendants will secure a higher style of order and embellishment than what exists in an ordinary cottage; for training, if it avails in nothing else, is equivalent to the appreciation of refinement in habits and manners, of personal neatness and tidiness, and to a knowledge of what comfort is.

These and similar details will naturally fall to be determined according to the views of the medical superintendent in each particular case. A very interesting table is given by Dr. Sibbald, in a paper upon the Cottage System and Gheel,* with a view to show approximately the number of patients out of 603 then in the Asylum at Morningside that might be regarded as suitable for a cottage, or suitable for a detached building, and the results are important, but involve considerations which cannot be entered upon here. It is likewise necessary to avoid the discussion of the advantages which might accrue from many such cottages as places of observation, and for the reception of certain classes of cases from without, or from extending the system to the affluent, who might continue in the bosom of their family while under the treatment of experts, and under that discipline which is found to be so powerful an adjuvant to physical means; or from seeking, in the opportunities for classification and minute subdivision which it affords, an escape from some of the difficulties which obstruct the introduction of individuals labouring under dipsomania, puerperal mania, and other forms of transitory mental disease within the outer cordon of a sanatorium.

* *The Journal of Mental Science*, April, 1861, p. 57.

